# Coinfection and Genetic Characterization of Porcine Astrovirus in Diarrheic Piglets in China From 2015 to 2018

**DOI:** 10.3389/fvets.2020.00462

**Published:** 2020-08-14

**Authors:** Mingjun Su, Shanshan Qi, Dan Yang, Donghua Guo, Baishuang Yin, Dongbo Sun

**Affiliations:** ^1^Laboratory for the Prevention and Control of Swine Infectious Diseases, College of Animal Science and Veterinary Medicine, Heilongjiang Bayi Agricultural University, Daqing, China; ^2^Heilongjiang Province Cultivating Collaborative Innovation Center for the Beidahuang Modern Agricultural Industry Technology, Daqing, China; ^3^College of Animal Science and Technology, Jilin Agricultural Science and Technology University, Jilin, China

**Keywords:** PAstV, coinfection, genetic characterization, diarrhea, piglet

## Abstract

Porcine astrovirus (PAstV) is broadly distributed globally and exists as at least five distinct genotypes. PAstV, which was recently identified as an important pathogen of diarrhea in piglets, is widely distributed in China. However, few studies have investigated the coinfection and genetic characterization of PAstV in diarrheic piglets in China. In this study, 89 PAstV-positive samples were identified in 543 diarrhea samples in China from 2015 to 2018, of which 75.28% (67/89) were coinfected with three to five different porcine pathogens, while none were positive for PAstV only. Among the 543 diarrhea samples, statistical analysis showed that PAstV-induced diarrhea was potentially associated with coinfection of PEV (*p* < 0.01) and GARV (*p* < 0.01). Phylogenetic analysis showed that the 27 identified PAstV strains belong to three different genotypes and that PAstV-2 (81.48%, 22/27) was predominant in diarrheic piglets in China, followed by PAstV-4 (11.11%, 3/27) and PAasV-5 (7.41%, 2/27). Sequence analysis revealed that the 27 RdRp genes identified in this study had nucleotide homologies of 53.8–99.5%. In addition, the RdRp gene of PAstV-4 strain JL/MHK/2018/0115 harbored a unique insert of three nucleotides (GAA) as compared with other known PAstV-4 strains. Furthermore, the genotypes of PAstV varied among different geographical locations, although PAstV-2 was the most widely distributed in China. These data demonstrate that PAstV coinfection with other porcine pathogens was common and there was genetic diversity of PAstV in diarrheic piglets in China.

## Introduction

Porcine astrovirus (PAstV), belonging to the family *Astroviridae*, genus *Mamastrovirus*, is a non-enveloped, single-strand, positive-sense RNA virus (1). The PAstV genome consists of three open reading frames (ORFs): ORF1a, ORF1b, and ORF2. ORF1a and ORF1b encode non-structural proteins and the RNA-dependent RNA polymerase (RdRp), and ORF2 encodes the capsid protein (2). PAstV was first identified in diarrheic piglets in 1980 (1). Since then, PAstV has been isolated worldwide, including Europe, Asia, and the Americas (1, 3–6).

Diarrhea of piglets has long been a problem afflicting the global pig industry. Coinfections with more than one porcine pathogen are common and often more clinically severe (7, 8). A previous surveillance study conducted by our group found a high percentage of coinfection among diarrheic piglets (9). PAstV, which has been identified as an important agent of diarrhea (10), and frequently presents as a coinfection with other porcine pathogens (3, 5, 6, 9, 11). However, data regarding coinfections with PAstV in diarrheic piglets in China are limited. Therefore, coinfections with PAstV and other porcine pathogens should be monitored in China.

To date, five genotypes of PAstV (PAstV-1 to PAstV-5) with different prevalences have been identified worldwide. Although all five PAstV genotypes have been reported in Europe, the most common is reportedly PAstV-4 (4), while PAstV-2 and PAstV-4 are the most common throughout Asia (5, 6, 12). However, information available on the genetic characterization of PAstV in China is fairly limited (2, 11). Therefore, it is necessary to investigate the genetic diversity and evolution of PAstV currently in China.

In this study, 89 PAstV-positive diarrhea samples were collected to investigate the prevalence of PAstV coinfection with 12 other porcine pathogens. The obtained RdRp genes were genetically characterized in order to provide insights into the epidemiology of PAstV circulating among diarrheic piglets in China.

## Materials and Methods

### Sample Collection

In our previous study (9), 89 (16.4%) of 543 diarrhea samples collected from 17 provinces or municipalities in China (Anhui, Fujian, Guangdong, Hebei, Heilongjiang, Hubei, Hunan, Jiangxi, Jilin, Liaoning, Shandong, Shaanxi, Shanxi, Sichuan, Shanghai, and the Inner Mongolia Autonomous Region; Table S1) from 2015 to 2018 were confirmed as PAstV-positive by reverse-transcription polymerase chain reaction (RT-PCR) and stored at −80°C.

### Sequencing and Analysis of the RdRp Gene of PAstV

RNA extraction and cDNA synthesis were performed as previously described by Wang et al. (13). The PAstV RdRp gene was amplified using the nested RT-PCR method described by Chu et al. (14) and then cloned into the vector pMD18-T (TaKaRa Biotechnology Co., Ltd, Dalian, China) in accordance with the manufacturer's protocol. Three positive clones of each amplicon were subjected to Sanger sequencing. Sequence analysis was conducted using the EditSeq tool included with the Lasergene DNASTAR™ 5.06 software package (DNASTAR Inc., Madison, WI, USA). Multiple-sequence alignments were performed using the multiple-sequence alignment tool included with the DNAMAN 6.0 software package (Lynnon BioSoft, Pointe-Claire, QB, Canada).

### Phylogenetic Analysis

Sequences of the PAstV RdRp gene retrieved from the GenBank database (https://www.ncbi.nlm.nih.gov/genbank/) were used for sequence alignments and phylogenetic analyses. Multiple-sequence alignments were generated using the ClustalX alignment program included with the MEGA 6.06 software package (15). A phylogenetic tree was constructed from the aligned nucleotide sequences using the p-distance model and 1000 bootstrap replicates and annotated with Interactive Tree Of Life (iTOL) software (http://itol.embl.de/) (16).

### Statistical Analysis

The correlation of PAstV infection with other pathogens was assessed with the use of 2 × 2 contingency tables and the chi-square (χ^2^) test with confidence limits of 95%. All analyses were performed using IBM SPSS Statistics for Windows, version 22.0 (IBM Corporation, Armonk, NY, USA). Probability (*p*) values of <0.05 and 0.01 were considered statistically significant and highly significant, respectively. Data regarding the detection of porcine circovirus type 3, porcine group A rotavirus (GARV), mammalian reovirus, porcine bocavirus, porcine deltacoronavirus, porcine enterovirus 9/10 (PEV), porcine kobuvirus (PKV), porcine sapelovirus, porcine torovirus, porcine teschovirus, porcine transmissible gastroenteritis virus (TGEV), and torque teno sus virus 2 in the 543 diarrhea samples (including the 89 PAstV-positive samples) were published in our previous reports (9, 17).

## Results and Discussion

### Coinfection of PAstV With Multiple Porcine Pathogens in Diarrheic Piglets

**Figure 1:**
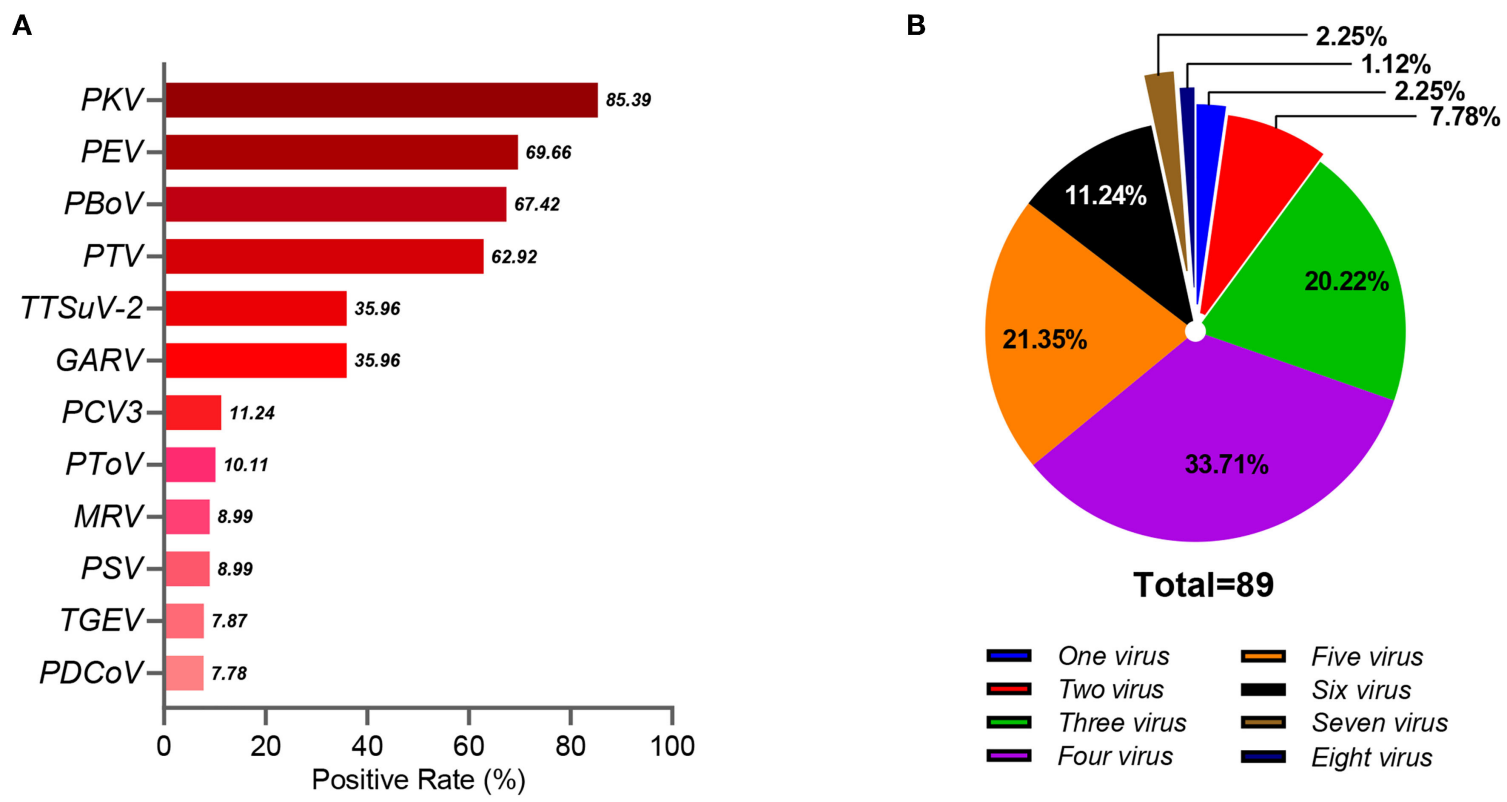
The coinfection of PAstV with multiple porcine pathogens in diarrheic piglets. **(A)** The positive rate of the 12 porcine pathogens in the 89 PAstV-positive samples. **(B)** The coinfection patterns of the 12 porcine pathogens in the 89 PAstV-positive samples.

As reported in our previous study, the PAstV-positive rate in diarrheic piglets was 16.39% (89/543), indicating wide distribution in China (9). In the present study, PAstV coinfection with 12 other porcine pathogens in diarrheic piglets was investigated. Of the 89 PAstV-positive diarrhea samples, the rate of PAstV coinfection with 12 other porcine pathogens ranged from 7.87% (7/89) to 85.39% (76/89) (Figure 1A). The average number of viruses detected in each sample was 4.12, while 75.28% (67/89) of samples had three to five different viruses, 3.37% (3/89) had seven to eight different viruses, and none were positive for PAstV only (Figure 1B). Coinfections of PAstV with other porcine pathogens, such as rotavirus, PEDV, TGEV, porcine circovirus-2 (PCV2), and porcine hemagglutinating encephalomyelitis virus (PHEV), have been reported previously (3, 5, 6, 11). In the present study, the rate of PKV coinfection in PAstV-positive samples was relatively high (85.39%, 76/89), and there was evidence that u shed more PKV than did healthy individuals (18), indicating that PKV may have a potential role in PAstV-induced diarrhea in piglets. PEDV is the major cause of viral diarrheal disease in swine in China (9). Although there was a high prevalence of PEDV in piglets infected with PAstV in our previous study, statistical analysis indicated that PEDV-induced diarrhea was not associated with PAstV coinfection (*p* > 0.05) (9). The pathogenicity of PEV is typically mild in pigs (19), and GARV is among the most common pathogens of diarrhea in piglets (20). In this study, the positivity rates of PEV (69.66%, 62/89) and GARV (35.96%, 32/89) were relatively high in PAstV-positive samples, suggesting a highly significant association of coinfection with PEV (*p* < 0.01) and GARV (*p* < 0.01) in PAstV-positive diarrhea samples of piglets (Table 1). However, since evidence of coinfections of PAstV with PEV and GARV causing diarrhea in piglets is somewhat limited, further studies are warranted.

**Table 1:** The statistical analysis of correlations of PAstV with other porcine pathogens.

	***P*-value**	**Odds ratio (OR)**	**95% confidence interval (95% CI)**
PKV	0.103	1.778	0.908–3.480
PEV	0.000	4.219	2.582–6.896
PBoV	0.193	1.384	0.855–2.240
PTV	0.722	1.115	0.697–1.783
TTSuV-2	0.623	1.149	0.715–1.848
GARV	0.000	2.563	1.563–4.246
PCV3	0.702	1.150	0.556–2.379
PToV	0.393	1.390	0.642–3.009
MRV	0.583	0.747	0.342–1.631
PSV	0.128	1.939	0.835–4.507
TGEV	1.000	0.908	0.393–2.101
PDCoV	0.182	1.852	0.759–4.522

*OR, odd ratio; CI, confidence interval*.

### Phylogenetic Analysis of PAstV

**Figure 2:**
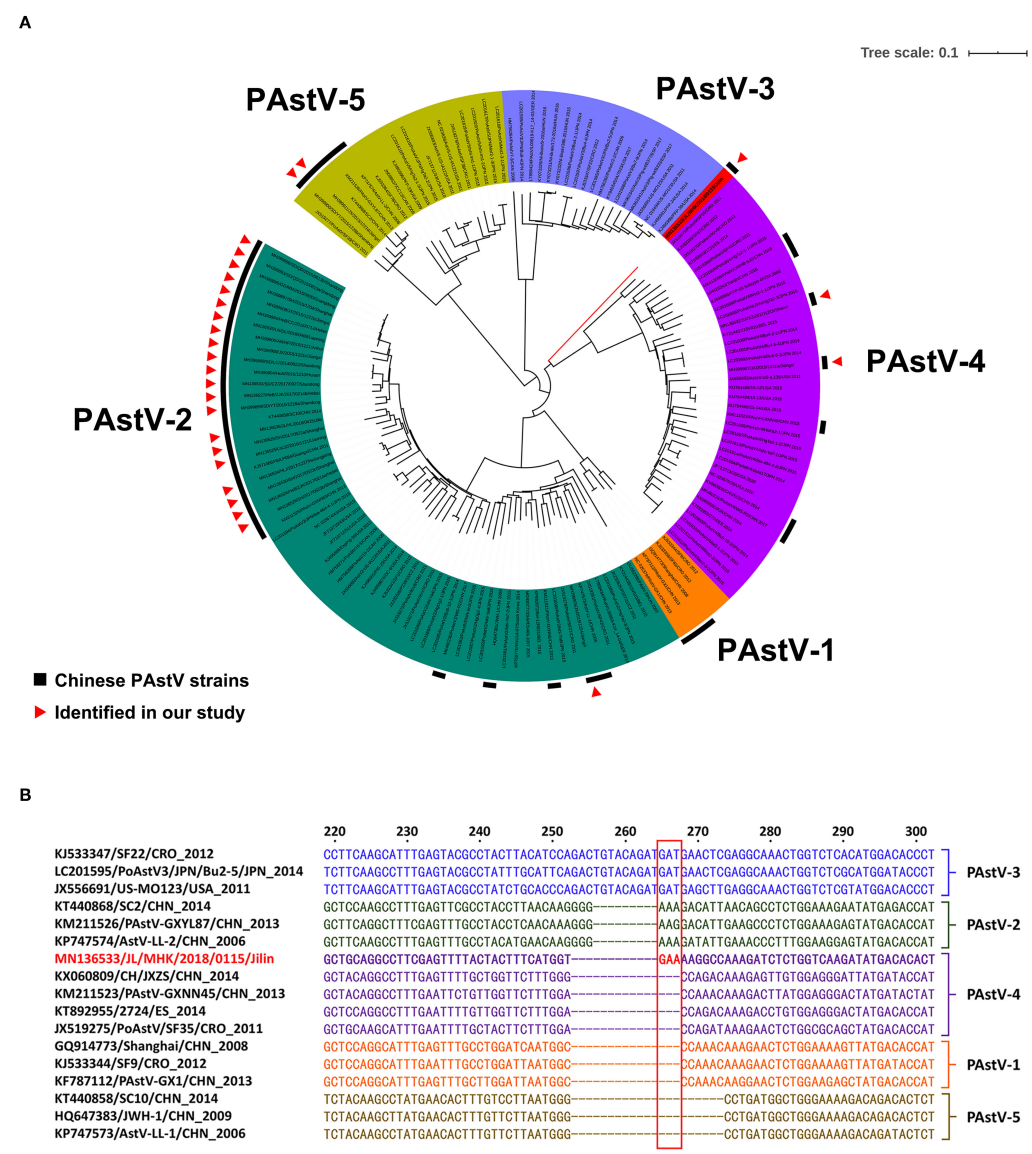
**(A)** The tree was constructed on the base of 366- to 381-bp (depend on PAstV genotype) nucleotide sequences of RdRp of the PAstV and selected sequences from GenBank. **(B)** Sequence analysis of the RdRp gene of the PAstV strain JL/MHK/2018/0115.

In the current study, a total of 27 RdRp genes were successfully sequenced from the 89 PAstV-positive diarrhea samples (Table S2). The RdRp gene is widely used to classify the genotype of PAstV (5, 11, 12, 21–23). Here, a phylogenetic tree was constructed based on the RdRp genes of the 27 identified PAstV strains and 115 reference PAstV strains. In the phylogenic tree, 142 PAstV strains were divided into five groups and three distinct genotypes (PAstV-2, PAstV-4, and PAstV-5) (Figure 2A). In the phylogenetic analysis, 22 identified PAstV strains and 39 reference strains from nine other countries were placed into the PAstV-2 group and divided into two clusters. With the exception of PAstV strain JX/2015/1224, all other identified PAstV strains and 21 reference PAstV strains formed one cluster in the PAstV-2 group, which shared nucleotide homologies of 84.8–99.5%. PAstV strain JX/2015/1224 and 18 reference PAstV strains shared nucleotide identities of 83.1–90.2% and formed the other cluster in the PAstV-2 group. Two identified PAstV strains, SD/YT/2015/1228b and JX/2015/1221d, which are closely related to a PAstV strain isolated in Croatia, were classified into the PAstV-5 group, which had nucleotide identities of 79.8–94.0%. Three identified PAstV strains (JL/MHK/2018/0115, SX/XZ/2017/1215, and JX/2015/1221a) and 35 reference strains, which shared nucleotide homologies of 62.1–93.6%, were classified to the PAstV-4 group. Most of the 27 identified PAstV strains were classified to the PAstV-2 group (81.5%, 22/27), indicating that PAstV-2 was predominant in diarrheic piglets in China. Similarly, the high prevalence of PAstV-2 in China was reported by Cai et al. (11) and Qin et al. (22). In contrast, the prevalence of PAstV-4 is reportedly higher than that of PAstV-2 in Thailand, South Korea, and India (5, 6, 12).

Sequence analysis of the 27 RdRp genes identified in this study had nucleotide identities of 53.8–99.5%, indicating a wide variation at the nucleotide level. PAstV strain JL/MHK/2018/0115 differed genetically from other PAstV-4 strains and formed a unique clade within the PAstV-4 group. Sequence analysis showed that the RdRp gene of strain JL/MHK/2018/0115 had low sequence similarity to the other PAstV-4 strains, with nucleotide identities of 62.1–67.1% and had a unique insert of three nucleotides (GAA) as compared with other PAstV-4 strains (Figure 2B). Previous studies have reported a high prevalence of PAstV-4 in other Asian countries, including South Korea (88.46%, 23/26), Thailand (92.00%, 23/25), and India (95.65%, 22/23), as well as European countries (70.4%, 295/419) (4–6, 12). PAstV-4, which was first reported in 2013, is a newly identified genotype in China that has since spread to the provinces of Hunan, Tianjin, Shanxi, Jilin, and Jiangxi (9, 21, 22). In addition, Lv et al. (21) and Zhao et al. (24) reported novel recombinant PAstV-4 strains in China. These data suggest that the Chinese PAstV-4 strains have undergone genetic variations and may have become the predominant strains in diarrheic piglets in China.

### Genotype Distribution of PAstV in China

**Figure 3:**
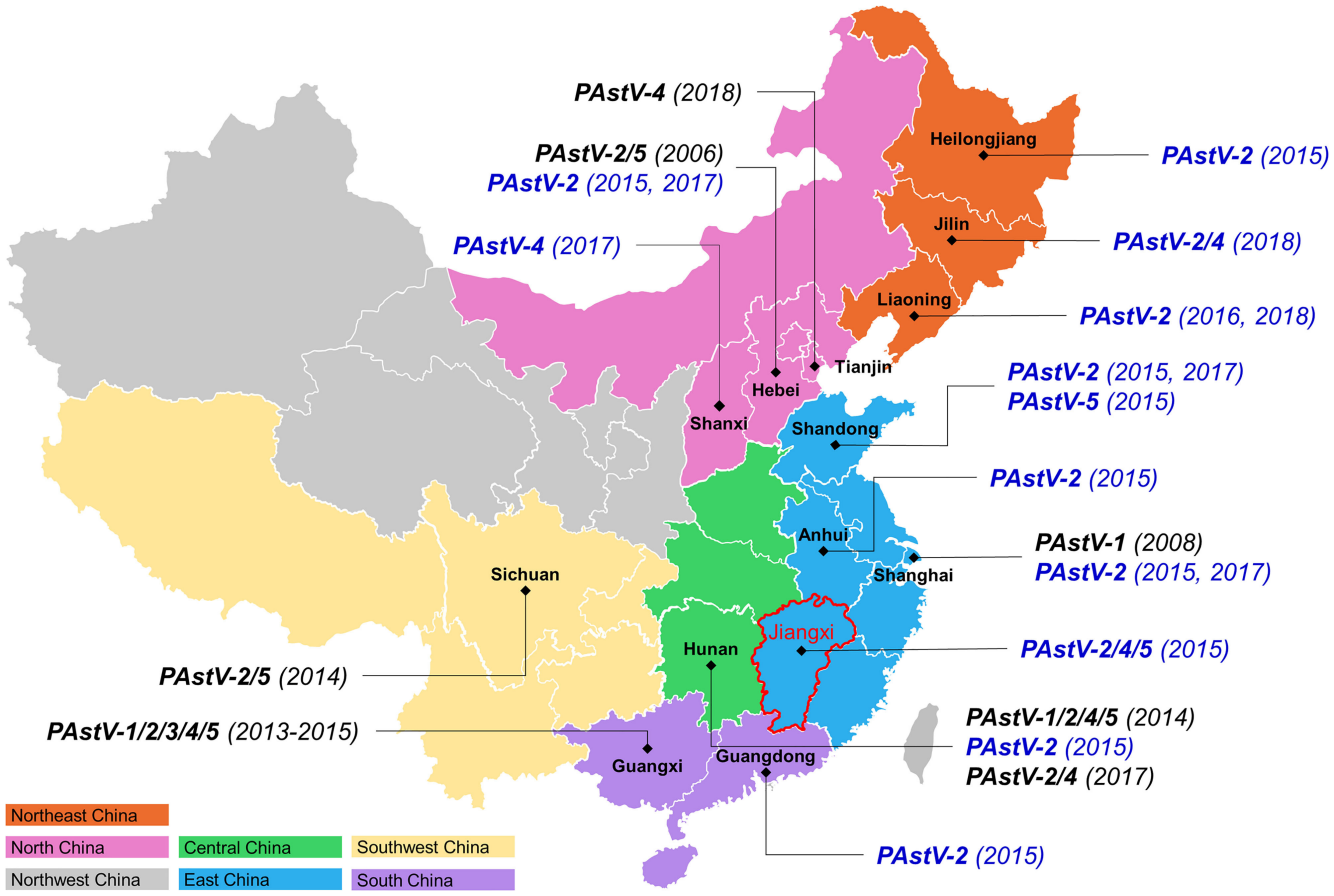
Genotype distribution of PAstV strains in China. The 27 identified PAstV strains were shown in blue. The numbers in bracket are the collection date of samples.

The results of the present study showed that PAstV-2 was circulating in ten different provinces, covering five regions of China (Figure 3), suggesting that PAstV-2 is the most widely distributed strain in China, which is supported by recent studies conducted in the provinces of Hebei, Hunan, Sichuan, and Guangxi (11, 21, 22, 25). Previous studies have reported the existence of multiple PAstV genotypes in China. For example, PAstV-1 was identified in Shanghai as well as the provinces of Hunan and Guangxi (22, 25), while PAstV-3 was identified in Guangxi province only (22), PAstV-4 has been reported in the provinces of Tianjin, Hunan, and Guangxi (22, 24, 25), and PAstV-5 has been detected in the provinces of Hebei, Sichuan, Hunan, and Guangxi (11, 22, 24–26). In the present study, three PAstV-4 strains were identified in the provinces of Jilin, Shanxi, and Jiangxi, and two PAstV-5 strains were identified in Shandong and Jiangxi provinces, respectively. These results suggest the presence of various genotypes of PAstV in different regions of China. Moreover, five PAstV strains identified in Jiangxi province exhibited three distinct genotypes (PAstV-2, PAstV-4, and PAstV-5). Previous studies reported the presence of two or more genotypes of PAstV in the same province of China, such as four different genotypes of PAstV circulating in Hunan province (21, 25), while all five genotypes of PAstV were identified in Guangxi province (22), indicating a remarkable diversity of genotypes of PAstV cocirculating among pig farms in China.

In conclusion, results from the present study provide evidence that coinfection of PAstV with multiple porcine pathogens is common in diarrheic piglets in China, and PAstV-induced diarrhea is potentially associated with PEV and GARV coinfection. Phylogenetic analysis revealed that PAstV-2 was predominant in diarrheic piglets in China and multiple genotypes of PAstV were co-circulating in China from 2015 to 2018. In addition, one PAstV-4 strain was shown to harbor a unique insert within the RdRp gene. These results increase our current understanding of the coinfection and genetic characterization of PAstV in diarrheic piglets in China and provide valuable information for further studies of PAstV.

## Data Availability Statement

The datasets presented in this study can be found in online repositories. The names of the repository/repositories and accession number(s) can be found in the article/Supplementary Material.

## Ethics Statement

This animal study was reviewed and approved by Animal Experiments Committee of the Heilongjiang Bayi Agricultural University (registration protocol 201501003). Written informed consent was obtained from the owners for the participation of their animals in this study.

## Author Contributions

DS conceived the study. MS, SQ, DY, DG, and BY analyzed the data. MS and SQ wrote the manuscript. All authors contributed to the article and approved the submitted version.

## Conflict of Interest

The authors declare that the research was conducted in the absence of any commercial or financial relationships that could be construed as a potential conflict of interest.

## Supplementary Material

Table S1 The background information of 89 PAstV-positive samples.

Table S2 The PAstV RdRp genes identified in this study.
