# Supplementary material for: Coinfection and Genetic Characterization of Porcine Astrovirus in Diarrheic Piglets in China From 2015 to 2018
Source: Front Vet Sci. 2020 Aug 14;7:462. doi: 10.3389/fvets.2020.00462 (PMC7456941; doi:10.3389/fvets.2020.00462)
Supplement: Table S2 — The PAstV RdRp genes identified in this study. [file Table_2.DOC]

**Table S2. The PAstV RdRp genes identified in this study.**

| **Strain name** | **Geographic location (Province)** | **Collection date** | **GenBank accession no.** |
| --- | --- | --- | --- |
| SD/QD/2015/0613b | Shandong | 06-2015 | MH399893 |
| HeB/CC/2015/0714 | Hebei | 07-2015 | MH399894 |
| GD/MM/2015/0820 | Guangdong | 08-2015 | MH399895 |
| SD/QD/2015/0613b | Shandong | 06-2015 | MH399896 |
| SH/2015/1204 | Shanghai | 12-2015 | MH399897 |
| SD/LC/2015/0822 | Shandong | 08-2015 | MH399898 |
| SD/YT/2015/1228a | Shandong | 12-2015 | MH399899 |
| SD/YT/2015/1228b | Shandong | 12-2015 | MH399900 |
| HuN/2015/1210 | Hunan | 12-2015 | MH399904 |
| JX/2015/1224 | Jiangxi | 12-2015 | MH399905 |
| AH/HF/2015/1221 | Anhui | 12-2015 | MH399906 |
| JX/2015/1221a | Jiangxi | 12-2015 | MH399907 |
| JX/2015/1221b | Jiangxi | 12-2015 | MH399908 |
| JX/2015/1221c | Jiangxi | 12-2015 | MH399909 |
| JX/2015/1221d | Jiangxi | 12-2015 | MH399911 |
| LN/JZ/2016/1221 | Liaoning | 12-2016 | MN136525 |
| HeB/ZJK/2017/0214a | Hebei | 02-2017 | MN136526 |
| HeB/ZJK/2017/0214b | Hebei | 02-2017 | MN136527 |
| SH/2017/0523a | Shanghai | 05-2017 | MN136528 |
| SH/2017/0523b | Shanghai | 05-2017 | MN136529 |
| SH/2017/0523c | Shanghai | 05-2017 | MN136530 |
| SD/CZ/2017/0927 | Shandong | 09-2017 | MN136531 |
| SX/XZ/2017/1215 | Shanxi | 12-2017 | MN136532 |
| JL/MHK/2018/0115 | Jilin | 01-2018 | MN136533 |
| HLJ/2017/1227 | Heilongjiang | 12-2017 | MN136534 |
| LN/DL/2018/0409 | Liaoning | 04-2018 | MN136535 |
| JL/HL/2018/0415 | Jilin | 04-2018 | MN136536 |
